# Concentration of testosterone glucuronide in urine from women with breast tumours.

**DOI:** 10.1038/bjc.1977.133

**Published:** 1977-06

**Authors:** M. K. Jones, I. D. Ramsay, W. P. Collins


*Br. J. Cancer* (1977) **35**, 885.

### *Short Communication*

### CONCENTRATION OF TESTOSTERONE GLUCURONIDE IN URINE FROM WOMEN WITH BREAST TUMOURS§

M. K. JONES,* I. D. RAMSAY† AND W. P. COLLINS‡

*From the *Faith Courtauld Unit for Human Studies in Cancer, King's College Hospital Medical School, London SE5 8RX, †the Regional Endocrine Centre, North Middlesex Hospital, London N18, and ‡the Department of Obstetrics and Gynaecology, King's College Hospital Medical School, London SE5 8RX*

Received 26 October 1976 Accepted 17 January 1977

THERE is a great deal of indirect evidence that the ovaries are involved in the aetiology of breast cancer. Thus normal ovarian function and pregnancy are considered to exert some protective effect against the development of breast tumours (Lowe and MacMahon, 1970). In contrast, sterile women have a higher risk factor and anovulation is present in a large number of patients with the established disease (Grattarola, 1964). Furthermore, ovarian dysfunction in patients with breast cancer is often accompanied by endometrial hyperplasia and marked increases in the level of urinary testosterone (Grattarola, 1967, 1969). It has also been shown that these patients have significantly higher levels of the metabolite than women with endometrial hyperplasia alone—both before and after the menopause (Grattarola, 1967, 1970; Grattarola *et al.*, 1974). It was postulated that the increased androgen production probably originated from the ovaries. This claim is supported by the finding of interstitial-cell hyperplasia (Hall and Dederiel, 1959), and the conclusion that this ovarian compartment is concerned primarily with the production of $C_{19}$-steroids (Rice and Savard, 1966; Mattingley and Huang, 1969).

The aim of the present study was to obtain further information on the concentration of testosterone glucuronide in urine from matched groups of women with either benign or malignant tumours of the breast.

Eighty-nine patients (aged 25–70 years) were studied after being admitted to hospital for breast surgery. All gave their informed consent. After the tissue had been removed the women were divided into two groups depending on whether the tumour was benign or malignant according to the histology report. Only patients with cancer Stages I and II of the Manchester classification (Wise, York Mason and Ackerman, 1971) were included in the group with malignancies. The subjects were then categorized as being either pre- or postmenopausal, depending on whether 6 months or more had elapsed since the last menstrual period. The menopausal status of every subject was checked 2 years later, and if any doubt existed about their original classification, these patients were excluded from the study. Similarly, patients on drugs likely to affect androgen metabolism were not included in the investigation.

Urine samples were collected for 24 h from every patient from Days 3 to 2 prior to the operation. The concentration of testosterone glucuronide was measured directly by radioimmunoassay without hydrolysis or extraction. Fifty $\mu$l of urine was dried at 100°C to eliminate non-specific binding by urinary proteins.

§ This work was carried out on behalf of the Faith Courtauld Unit for Human Studies in Cancer.

The residues were cooled, resdissolved in buffer and equilibrated with antiserum to testosterone-17$\beta$-glucosiduronyl-bovine serum albumin and tritiated testosterone glucuronide. The unbound steroid was removed with dextran-coated charcoal. The method has been described and evaluated in detail (Hennam, Collins and Sommerville, 1973). The phase of the menstrual cycle was not taken into consideration in those women who still had menses, because the fluctuations in the amount of metabolite excreted are small and the pattern is variable (Collins and Hennam, 1976).

The frequencies with which various concentrations of testosterone glucuronide appeared in each group were studied, and it was concluded that comparisons were best carried out using a non-parametric method, *viz*: the Mann-Whitney U Test. The results are shown in the Table.

TABLE.—*The Concentration (nmol/24 h) of Testosterone Glucuronide in Urine from Women with Benign or Early Malignant Disease of the Breast.*

| Source | No. of subjects | Median | Range |
|---|---|---|---|
| Premenopausal | | | |
| Benign | 40 | 48·42 | 15·61–110·06 |
| Cancer | 16 | 46·91 | 8·77–119·03 |
| Postmenopausal | | | |
| Benign | 8 | 34·08 | 17·46–87·95 |
| Cancer | 25 | 31·31 | 17·26–98·31 |

There were no significant differences in the concentration of testosterone glucuronide in samples from patients with benign or malignant breast disease—either before or after the menopause. This finding is in agreement with a previous study on the concentration of testosterone in peripheral venous plasma from the same groups of subjects (Jones *et al.*, 1977). The results, however, do not concur with those of Grattarola, who measured total urinary testosterone by gas-liquid chromatography, in patients with early cancer of the breast, and found higher values than in a healthy control group (Grattarola, 1967, 1970, 1973; Grattarola *et al.*, 1974). Accordingly, our data do not support the rationale for offering ovarian ablation therapy to postmenopausal patients with breast cancer, when increased androgen production is proven (Hall and Dederiel, 1959), neither does it help to explain the claim that early oophorectomy significantly reduces the incidence of breast cancer (Feinleib, 1968)

There is, however, much evidence of disturbed androgen metabolism in women with breast cancer. For example, there is a decreased level of androsterone and aetiocholanone in urine (Hayward and Bulbrook, 1968; Bulbrook, Hayward and Spicer, 1971). These metabolites are derived from circulating dehydroepiandrosterone sulphate, dehydroepiandrosterone and androstenedione, and are thus primarily of adrenal origin. In addition, there is an increased production of sebum by the sebaceous glands, which are androgen-sensitive (Burton, Cunliffe and Schuster, 1970). Testosterone glucuronide in urine is derived from circulating testosterone (~0·1%), androstenedione (0·4%) and dehydroepiandrosterone (0·003%). It is thus of adrenal and ovarian origin, and is a more informative index concerning the production of androgens, according to the standard definition (Sommerville and Collins, 1970). Thus the findings to date suggest that it is the weaker androgens of adrenal origin that are more concerned in the aetiology of breast cancer, or perhaps it is small differences in the level of biologically active androgen at the level of the responsive tissue. It is apparent that more detailed information is required on androgen metabolism in these patients.

### REFERENCES

BULBROOK, R. D., HAYWARD, J. L. & SPICER, C. C. 1971 Relation Between Urinary Androgen and Corticoid Excretion and Subsequent Breast Cancer. *Lancet*, ii, 395.

BURTON, J. L., CUNLIFFE, W. J. & SCHUSTER, S. (1970) Increased Sebum Secretion in Patients with Breast Cancer. *Br. med. J.*, i, 665.

COLLINS, W. P. & HENNAM, J. F. (1976) Methods for the Measurement of Gonadal Steroids and their Metabolites. Radioimmunoassay and Reproductive Endocrinology. In *Molecular Aspects of*

*Medicine*. Eds. H. Baum & J. Gergely. Oxford: Pergamon Press Ltd. p. 55.

FEINLEIB, M. F. (1968) Breast Cancer and Artificial Menopause: a Cohort Study. *J. natn. Cancer Inst.*, **41**, 315.

GRATTAROLA, R. (1964) The Premenstrual Endometrial Pattern of Women with Breast Cancer. *Cancer, N.Y.*, **17**, 1119.

GRATTAROLA, R. (1967) Urinary Excretion of 11-deoxy-17-ketosteroids by Patients with Breast Cancer with or without Ovulatory Cycles. *Endocrinology*, **38**, 77.

GRATTAROLA, R. (1969) Misdiagnosis of Endometrial Carcinoma in Young Women with Polycystic Ovarian Disease. *Am. J. Obst. Gyn.*, **105**, 498.

GRATTAROLA, R. (1970) Alteration of Malignant Processes by Hormonal Manipulation. *X Int. Cancer Congress Oncology*, **1**, 345.

GRATTAROLA, R. (1973) Androgens in Breast Cancer. Atypical Endometrial Hyperplasia and Breast Cancer in Married Premenopausal Women. *Am. J. Obst. Gyn.*, **116**, 423.

GRATTAROLA, R., SECRETO, G., RECCHIONE, C. & CASTELLINI, W. (1974) Androgens in Breast Cancer. *Am. J. Obst. Gyn.*, **118**, 173.

HALL, T. C. & DEDERIEL, M. M. (1959) *Acta. Un. Int. Cancer*, **15**, 1099.

HAYWARD, J. L. & BULBROOK, R. D. (1968) Urinary Steroids and the Progress of Breast Cancer. In: *Prognostic Factors in Breast Cancer*. Ed. A. P. M. Forrest and R. B. Kunkler. Edinburgh: E. & S. Livingstone. p. 383.

HENNAM, J. F., COLLINS, W. P. & SOMMERVILLE, I. F. (1973) Radioimmunoassay of Urinary Testosterone Glucuronoside. *Steroids*, **21**, 285.

JONES, M. K., RAMSAY, I. D., COLLINS, W. P. & DYER, G. (1977) Plasma Testosterone Concentrations in Patients with Breast Cancer. *Eur. J. Cancer* (in press).

LOWE, C. R. & MACMAHON, B. (1970) Breast Cancer and Reproductive History of Women in South Wales. *Lancet*, i, 153.

MATTINGLEY, R. F. & HUANG, W. Y. (1969) Steroid Genesis of the Menopausal and Postmenopausal Ovary. *Am. J. Obst. Gyn.*, **103**, 679.

RICE, B. E. & SAVARD, K. (1966) Steroid Hormone Formation in the Human Ovary: Ovarian Stromal Compartments; Formation of Radioactive Steroids from Acetate-I-$C^{14}$ and Action of Gonadotrophins. *J. clin. endocr. Meth.*, **26**, 593.

SOMMERVILLE, I. F. & COLLINS, W. P. (1970) Indices of Androgen Production in Women. In: *Advances in Steroid Biochemistry and Pharmacology*, Vol. 2. Ed. M. H. Briggs. London and New York: Academic Press. p. 267.

WISE, L., YORK MASON, A. & ACKERMAN, L. B. (1971) Local Excision and Irradiation: An Alternative Method for the Treatment of Early Mammary Cancer. *Ann. Surg.*, **174**, 392.


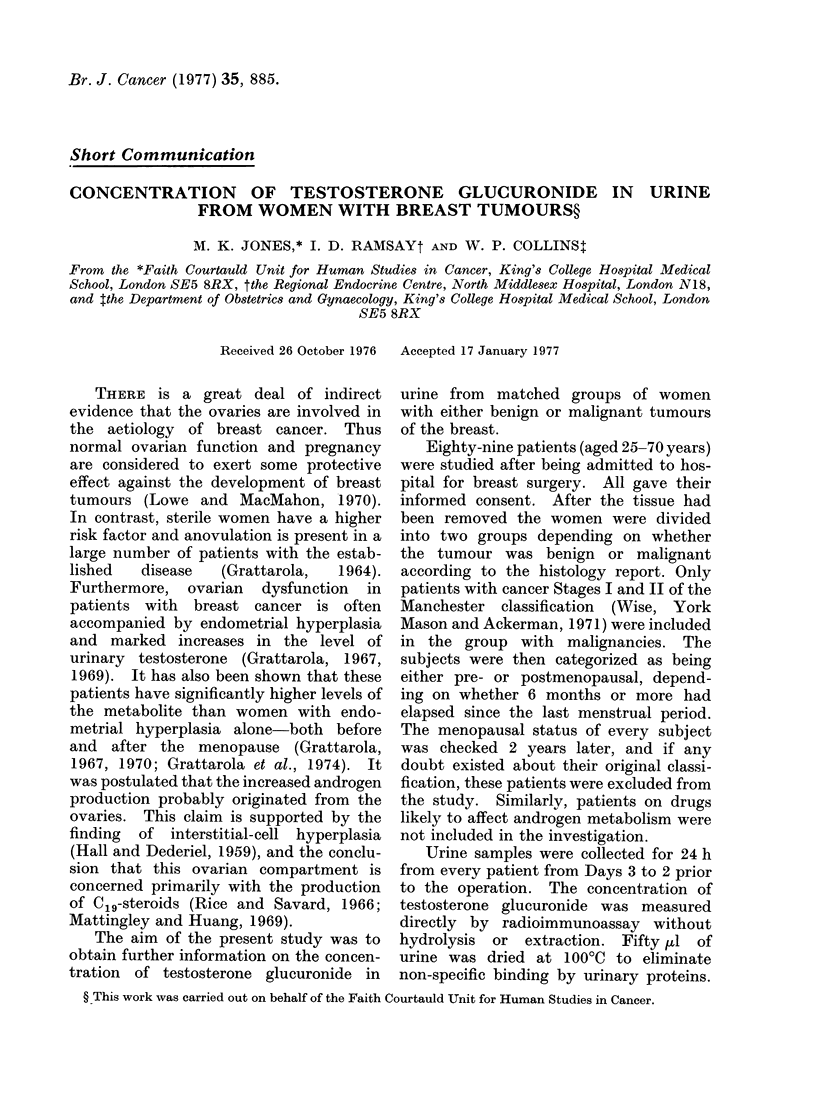

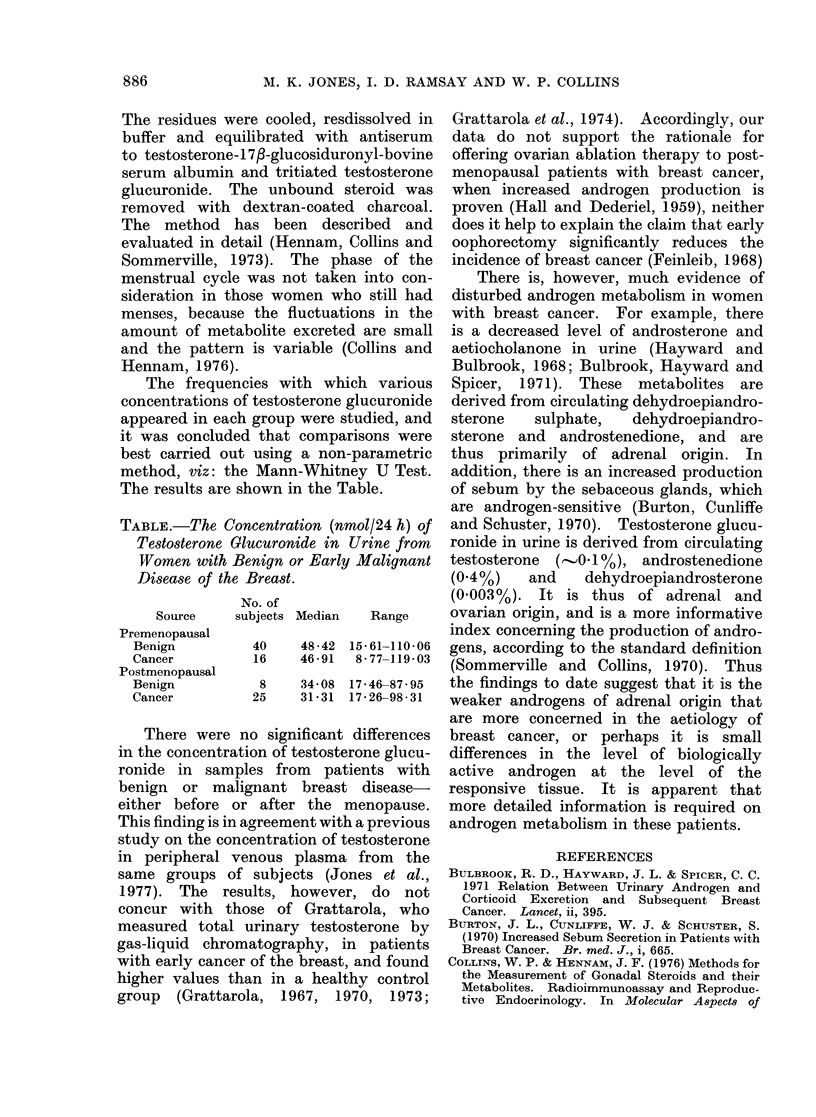

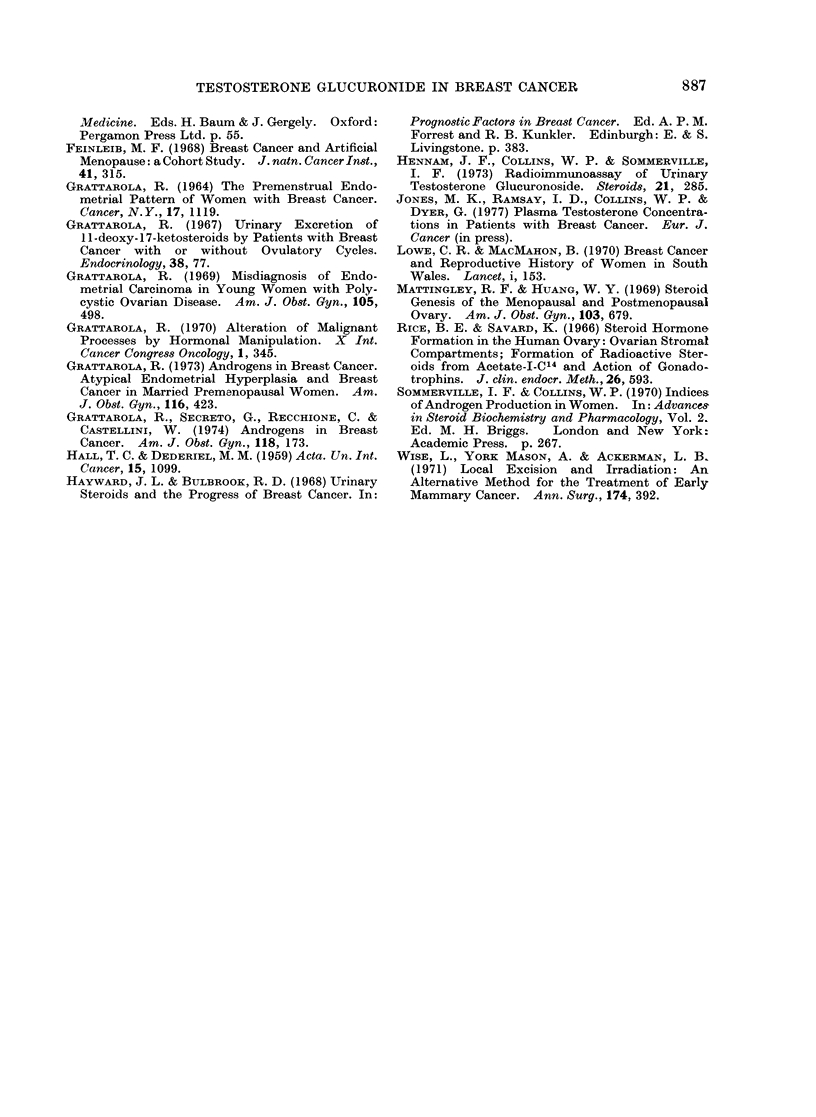


## References

[OCR_00203] Bulbrook R. D., Hayward J. L., Spicer C. C. (1971). Relation between urinary androgen and corticoid excretion and subsequent breast cancer.. Lancet.

[OCR_00209] Burton J. L., Cunliffe W. J., Shuster S. (1970). Increased sebum excretion in patients with breast cancer.. Br Med J.

[OCR_00225] Feinleib M. (1968). Breast cancer and artificial menopause: a cohort study.. J Natl Cancer Inst.

[OCR_00230] GRATTAROLA R. (1964). THE PREMENSTRUAL ENDOMETRIAL PATTERN OF WOMEN WITH BREAST CANCER. A STUDY OF PROGESTATIONAL ACTIVITY.. Cancer.

[OCR_00241] Grattarola R. (1969). Misdiagnosis of endometrial adenocarcinoma in young women with polycystic ovarian disease. Report of a case with an endocrine study.. Am J Obstet Gynecol.

[OCR_00258] Grattarola R., Secreto G., Recchione C., Castellini W. (1974). Androgens in breast cancer. II. Endometrial adenocarcinoma and breast cancer in married postmenopausal women.. Am J Obstet Gynecol.

[OCR_00235] Grattarola R. (1967). Urinary excretion of 11-deoxy-17-ketosteroids by patients with breast cancer with or without ovulatory cycles.. J Endocrinol.

[OCR_00285] Lowe C. R., MacMahon B. (1970). Breast cancer and reproductive history of women in South Wales.. Lancet.

[OCR_00295] Rice B. F., Savard K. (1966). Steroid hormone formation in the human ovary. IV. Ovarian stromal compartment; formation of radioactive steroids from acetate-1-14C and action of gonadotropins.. J Clin Endocrinol Metab.

[OCR_00309] Wise L., Mason A. Y., Ackerman L. V. (1971). Local excision and irradiation: an alternative method for the treatment of early mammary cancer.. Ann Surg.

